# Enhancing the maturity of *in vitro* engineered cartilage from Wharton's jelly-derived photo-crosslinked hydrogel using dynamic bioreactors and its *in vivo* outcomes in animal models

**DOI:** 10.1093/rb/rbaf037

**Published:** 2025-05-08

**Authors:** Chuanzhi Wei, Mingyue Lin, Qitao Bo, Wufei Dai, Jinghao Ding, Ru Chen

**Affiliations:** Department of Breast Surgery, Hainan Affiliated Hospital of Hainan Medical University (Hainan General Hospital), Haikou, 570311, China; Department of Breast Surgery, Hainan Affiliated Hospital of Hainan Medical University (Hainan General Hospital), Haikou, 570311, China; Department of Thoracic Surgery, Shanghai Pulmonary Hospital, Tongji University School of Medicine, Shanghai, 200433, China; Department of Plastic and Reconstructive Surgery, Shanghai Ninth People’s Hospital, Shanghai Jiao Tong University School of Medicine, Shanghai, 200011, China; Department of Breast Surgery, Hainan Affiliated Hospital of Hainan Medical University (Hainan General Hospital), Haikou, 570311, China; Department of Breast Surgery, Hainan Affiliated Hospital of Hainan Medical University (Hainan General Hospital), Haikou, 570311, China

**Keywords:** cartilage maturity, dynamic bioreactor, Wharton’s jelly, photo-crosslinking hydrogel, tissue engineering

## Abstract

The immature state of *in vitro* engineered cartilage (IVEC) hinders its clinical translation, highlighting the need for optimized scaffold platforms and cultivation models. Our previous work demonstrated that Wharton's jelly (WJ) contains an extracellular matrix (ECM) whose composition closely resembles that of native cartilage and includes several bioactive factors that promote chondrogenic induction. Furthermore, earlier studies have shown that photo-crosslinkable hydrogels are ideal carrier scaffolds for cartilage tissue engineering and that bioreactors improve nutrient and waste exchange between scaffolds and the culture medium. Based on these findings, we employed a dynamic bioreactor in combination with a WJ-derived photo-crosslinkable hydrogel to enhance IVEC maturity. Our results indicate that the decellularized WJ matrix (DWJM) effectively retains its native chondrogenic ECM components and bioactive factors. The photo-crosslinkable ADWJM hydrogel—produced by modifying DWJM with methacrylate anhydride—demonstrated excellent gelation capacity as well as tunable rheological properties, swelling ratios and degradation rates across different DWJM concentrations. In addition, the ADWJM hydrogel exhibited outstanding biocompatibility by providing a favorable 3D microenvironment for chondrocyte survival and proliferation. Most importantly, the dynamic bioreactor markedly promoted IVEC maturation. Constructs cultured under dynamic conditions displayed increased thickness, wet weight and volume; enhanced mechanical strength; more typical lacunae structures; and uniform deposition of cartilage-specific ECM compared to constructs maintained in static conditions or within a static bioreactor. Moreover, in vivo subcutaneous implantation of IVEC in goats further validated these findings, as the implanted constructs exhibited cartilage components and mechanical properties closely resembling those of natural cartilage. These results offer a promising approach for enhancing IVEC maturity and support its future clinical translation.

## Graphical abstract

**Figure:**
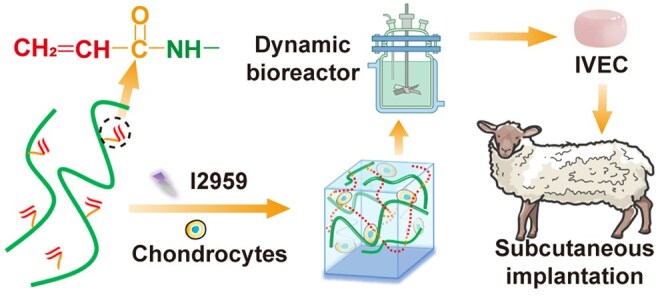

## Introduction

Cartilage defects are common in clinical practice and can result from inflammation, trauma, tumors or aging [1, 2]. Repairing cartilage in adults is challenging because the tissue is avascular, has a low cell-to-matrix ratio and possesses a dense extracellular matrix (ECM) rich in glycosaminoglycans (GAGs) and proteoglycans. These features severely limit cartilage’s intrinsic regenerative capacity [3]. Moreover, traditional repair methods such as autografts and allografts are restricted by donor availability, infection risks and immune rejection [4]. Consequently, there is an urgent need for novel therapeutic strategies.

Recent advances in cartilage tissue engineering have renewed interest in biomaterials and cell-based therapies. Two primary approaches are under investigation: (i) direct *in vivo* implantation of cell-loaded biomaterials and (ii) pre-cultivation of cell-loaded biomaterials to form *in vitro* engineered cartilage (IVEC) followed by subsequent *in vivo* implantation. Direct *in vivo* implantation faces several challenges. For instance, achieving seamless bonding with native cartilage is difficult, which often results in incomplete healing or poor interface attachment. Additionally, implanted biomaterials may trigger inflammation or rejection, and inadequate vascularization can restrict oxygen and nutrient delivery, thereby hindering cell survival and ECM formation. Furthermore, controlling cell differentiation and proliferation is challenging, increasing the risk of forming fibrocartilage instead of the desired hyaline cartilage. In contrast, IVEC exhibits histological characteristics and ECM components that closely resemble native cartilage. Since the cartilage is established *in vitro* prior to implantation, adverse factors—such as individual variations in bioactivity, cell loss, material-induced inflammatory responses and unfavorable local microenvironments—are minimized. Importantly, IVEC can be produced in a standardized manner and, if necessary, its implantation can be aborted without risk to the patient. These advantages offer a more reliable and clinically feasible approach for cartilage repair [5].

The formation of IVEC typically involves seeding chondrocytes onto suitable biomaterials and cultivating them under conditions enriched with chondrogenic factors or mechanical stimulation to promote functional cartilage formation [6, 7]. Hydrogels are particularly attractive for this purpose because they are highly hydrated 3D scaffolds that closely mimic the native cartilage ECM. Their excellent biocompatibility, tunable mechanical strength, adjustable degradation rates and capacity to incorporate bioactive molecules make them ideal for supporting cell adhesion, proliferation and differentiation. Moreover, the injectability of many hydrogel formulations facilitates the customization of irregular cartilage defects [8].

In recent years, Wharton’s jelly (WJ) has emerged as a promising natural material for regenerative medicine due to its biomimetic properties [9]. This mucous connective tissue surrounding the umbilical cord vessels is rich in ECM components—such as hyaluronic acid, various collagens and sulfated GAGs [10, 11]—and contains chondrogenic inductive factors like insulin-like growth factor (IGF), platelet-derived growth factor (PDGF) and transforming growth factor-beta1 (TGF-β1) [4, 12]. However, the cellular components in WJ can provoke immune reactions or potentially promote tumor formation. To overcome these issues, a decellularized WJ matrix (DWJM) has been developed that retains the native ECM and its bioactive factors while eliminating cellular elements [13]. Additionally, the mechanical properties of DWJM—capable of resisting compression and torsion—mirror those of cartilage, thereby reducing deformation, damage and friction. These attributes make DWJM a promising candidate for cartilage tissue engineering [14]. In this study, we aim to fabricate a photo-crosslinkable hydrogel derived from DWJM to promote the maturation of IVEC.

Despite early successes, hydrogel-based IVEC still suffers from significant limitations, such as poor mechanical properties and an immature ECM with low levels of cartilage-specific components [15]. These shortcomings likely result from static culture conditions that restrict nutrient uptake and metabolic activity. Biomechanical stimulation is critical for cartilage development and repair because it regulates chondrocyte proliferation, differentiation, gene expression, ECM deposition and nutrient exchange [16]. Bioreactors can simulate natural mechanical stresses—such as compression, tension and fluid shear stress—while maintaining a controlled environment for temperature, pH, oxygen, nutrients and growth factors [17]. Several studies have demonstrated that bioreactors enhance cartilage formation by improving biomechanical properties and increasing ECM deposition through effective material exchange between hydrogels and the culture medium [18, 19].

In our study, we first decellularized WJ using a NaOH solution to prepare DWJM and verified that it retained chondrogenic ECM components and bioactive factors. The DWJM was then acrylated to fabricate photo-crosslinkable ADWJM hydrogels with varying DWJM concentrations, and their physicochemical properties and biocompatibility were evaluated. Chondrocytes were seeded onto the optimized ADWJM hydrogel and cultured in a dynamic bioreactor for 8 weeks to assess IVEC maturity. For comparison, constructs were also maintained under static conditions using a static bioreactor and conventional culture methods. Finally, the matured IVEC was implanted subcutaneously in goats to further evaluate its capacity for cartilage formation *in vivo*. Collectively, our work presents a practical strategy for producing matured IVEC and supports its potential clinical application in cartilage defect repair.

## Materials and methods

### Preparation of DWJM

#### Decellularization of WJ matrix

Decellularization of WJ matrix was prepared according to the previously reported method [12]. Briefly, the umbilical cord WJ was carefully isolated with blood vessels and excess connective tissue was removed. The WJ was minced into approximately 0.5 × 0.5 cm fragments and placed in a 50 ml centrifuge tube, and 1 mol/l NaOH (Sigma-Aldrich, USA) solution was added. The mixture was incubated at room temperature for 3 hours for decellularization. After decellularization, the NaOH solution was removed, and the sample was washed with deionized water. The NaOH-treated WJ was then homogenized into a slurry using a tissue homogenizer at room temperature. The slurry was frozen overnight at –20°C and subsequently dried in a vacuum freeze-dryer for 48 hours to obtain WJ matrix foam. This foam was then digested with pepsin in an acetic acid solution (pH 2–3) at 37°C for 24 hours. Finally, the digested slurry was neutralized to pH 7.0 with NaOH, centrifuged (3000 rpm for 5 minutes), and freeze-dried to yield DWJM powder.

#### Gross appearance

For gross appearance analysis, samples from before and after decellularization (before any subsequent processing) were photographed. Their color and texture were recorded.

#### Scanning electron microscopy

Structural changes in the samples before and after decellularization were examined using scanning electron microscopy (SEM) (JEOL JSM-5600 LV, Japan) at 10 kV. Samples were fixed in 2.5% glutaraldehyde prepared in phosphate-buffered saline (PBS; Sigma-Aldrich, USA) and then gold-sputtered for 50 seconds to enhance conductivity. They were subsequently dehydrated using a graded ethanol series, dried to the critical point and imaged using the SEM apparatus [20].

#### Histological analysis

Samples before and after decellularization were fixed in 4% paraformaldehyde, embedded in paraffin and sectioned. Hematoxylin-eosin (H&E) staining was performed to observe morphological structure, while 4′,6-diamidino-2-phenylindole (DAPI; Sigma-Aldrich, USA) staining was used to visualize cellular components. Additionally, Safranin-O, Toluidine blue, Sirius red and Alcian blue stains were employed to evaluate GAGs, proteoglycans, collagen and chondroitin sulfate components, respectively.

#### Biochemical analysis

Cartilage-related biochemical components in the digested samples were quantitatively analyzed according to previously established methods [21]. DNA content was determined using the PicoGreen dsDNA assay (Sigma-Aldrich, USA), GAG levels were assessed with the dimethylmethylene blue (DMMB) assay (Sigma-Aldrich, USA), and hydroxyproline content was measured using a hydroxyproline assay kit (Sigma-Aldrich, USA). Additionally, bioactive factors—including PDGF, IGF-1, fibroblast growth factor-2 (FGF-2) and TGF-β1—were quantified using enzyme-linked immunosorbent assay (ELISA) kits.

### Preparation of photo-crosslinked ADWJM hydrogels

#### Preparation of ADWJM hydrogel precursors

ADWJM hydrogel precursor was prepared according to the previously reported method [22]. Briefly, 10 g of DWJM powder was dissolved and stirred in 500 ml of PBS (pH 7.4) at 50°C. Next, 10 ml of methacrylate anhydride was gradually added, and the reaction was allowed to proceed for 6 hours. The resulting solution was centrifuged at 5000 rpm for 10 minutes, and the supernatant was dialyzed against deionized water at 37°C for 3 days using a 14 kDa cut-off membrane. The dialyzed product was then lyophilized. Subsequently, the lyophilized ADWJM was reconstituted at different concentrations (5%, 10% and 15% w/v) with 0.3% w/v lithium phenyl-2,4,6-trimethylbenzoyl-phosphinate (LAP, a photo-initiator) in PBS. The final hydrogel precursors were stored at 4°C in the dark until further use.

#### ^1^H nuclear magnetic resonance spectroscopy

^1^H nuclear magnetic resonance (^1^H NMR) spectroscopy was used to characterize the structures of DWJM and the 10% ADWJM sample. Briefly, an appropriate amount of freeze-dried powder from each sample was dissolved in deuterated dimethyl sulfoxide (DMSO-d) at a concentration of 10 mg/ml. The solution was transferred to an NMR tube with tetramethylsilane (δ = 0.00 ppm) as the reference. Spectra were recorded on a 400 MHz Bruker NMR spectrometer, with chemical shifts reported in parts per million (ppm) and coupling constants (J) in Hertz (Hz) [22].

#### Gelation properties of ADWJM hydrogels

To assess gelation properties, 2 ml of ADWJM hydrogel precursor at concentrations of 5%, 10%, and 15% w/v were placed in bottles and irradiated with UV light (365 nm, 10 mW/cm^2^) for 10 minutes to induce polymerization. The process was recorded to document the gelation behavior for each group.

#### Rheological characterization of the ADWJM hydrogels

Rheological properties of ADWJM hydrogels at 5%, 10% and 15% w/v were measured using a controlled-stress rheometer equipped with a cone-plate geometry (2° cone angle, 20 mm cone diameter and a fixed gap of 0.105 mm). Dynamic temperature scanning was performed between 10 and 42°C at a rate of 1°C/min, and the storage modulus (G′) was recorded over 180 seconds [23].

#### Swelling ratio analysis of the ADWJM hydrogels

Hydrogels were cast into cylindrical shapes (4-mm diameter, 3-mm height) and freeze-dried to record their initial weight (W_1_). The samples were then immersed in 1 ml of sterile PBS (pH 7.4) at 37°C for 24 days. At predetermined time points, the samples were retrieved and reweighed (W_2_). The swelling ratio was calculated using the formula: (W_2_/W_1_) × 100%.

#### Degradation rate analysis of the ADWJM hydrogels

For degradation studies, ADWJM hydrogels (5%, 10% and 15% w/v) were cast into cylindrical shapes (4-mm diameter, 3-mm height) and placed in centrifuge tubes containing 15 ml of PBS with either 0 or 1.5 mg/l lysozyme (Sigma-Aldrich, USA). The tubes were incubated in a shaker at 37°C and 60 rpm, with PBS replaced daily. At specific intervals, samples were collected, and excess PBS was removed from the surface before measuring the swollen weight (W_t_). The samples were then freeze-dried to obtain the dry weight (W_d_). The degradation was quantified using the formula: [(W_t_−W_d_)/W_d_] × 100%.

### Biocompatibility assessment of ADWJM hydrogels

#### Isolation and culture of goat auricular chondrocytes

Auricular cartilage samples were obtained from three 8-month-old goats provided by the Experimental Animal Breeding Facility of Hainan Medical University (Haikou, Hainan Province). All animal procedures were approved by the Animal Care and Use Committee of Hainan Medical University (No. [2022]302). Cartilage samples were minced into approximately 1.0 mm^3^ fragments and digested with 0.15% type II collagenase (Gibco, USA) in Dulbecco’s Modified Eagle Medium (DMEM, Gibco, USA) at 37°C for 8 hours. The isolated chondrocytes were cultured in DMEM supplemented with 10% fetal bovine serum (FBS, Gibco, USA) and 1% penicillin-streptomycin (Gibco, USA) at 37°C in a 5% CO_2_ incubator. Passage 2 (P2) chondrocytes were used for subsequent *in vitro* cartilage evaluation [24].

#### Preparation of chondrocyte–hydrogel complexes

Chondrocyte suspensions at a concentration of 10 × 10^6^ cells/ml were prepared and mixed with hydrogels at 5%, 10% or 15% w/v. The mixtures were cast into custom-made cylindrical molds (10-mm diameter, 2-mm thickness) and exposed to 365 nm UV light (10 mW/cm^2^) for 10 minutes to achieve crosslinking and solidification. After removing the molds, the chondrocyte–hydrogel complexes were transferred to six-well plates containing 8 ml of DMEM supplemented with 10% FBS and 1% penicillin-streptomycin. The cultures were maintained at 37°C in a 5% CO_2_ incubator with medium changes every 2 days.

#### Biocompatibility assessment of the complexes

Chondrocyte–hydrogel complexes (5%, 10% and 15% w/v) were cultured at 37°C in a 5% CO_2_ incubator for 5 days. Live/dead cell viability staining (Invitrogen, USA) was performed on days 1, 3 and 5. Rhodamine-phalloidin was used to stain the cytoskeleton, and DAPI was used for nuclear staining. Stained samples were imaged using a confocal microscope (Nikon, Japan). Additionally, cell viability was assessed using a Cell Counting Kit-8 (CCK-8; Dojindo, Japan) following the manufacturer’s instructions. Optical density (OD) was measured at 450 nm, and the average OD from five wells was calculated. DNA content was quantified using the PicoGreen dsDNA assay kit as previously described [25].

### Maturity evaluation of IVEC under different culture systems

#### Mechanical stimulation modes

To evaluate IVEC maturity under different culture systems, chondrocyte–hydrogel complexes in the 15% group (prepared as described in the section Preparation of chondrocyte–hydrogel complexes) were randomly divided into three groups:

- Static culture group: complexes were cultured in six-well plates under static conditions at 37°C, 95% humidity and 5% CO_2_ without mechanical stimulation.
- Static bioreactor group: complexes were placed in a sealed bioreactor under constant pressure at 37°C, 95% humidity and 5% CO_2_, with daily stimulation at a constant pressure of 2 MPa for 30 minutes.
- Dynamic bioreactor group: complexes were subjected to dynamic compression at 37°C, 95% humidity, and 5% CO_2_ with gradually increasing pressure up to 2 MPa followed by gradual decompression in a continuous cyclic manner. This procedure was applied daily for 30 minutes.

#### Gross appearance

After 4 weeks of culture under the different mechanical stimulation modes, IVEC samples were harvested. Their gross appearance—including shape, color, texture and elasticity—was recorded and evaluated macroscopically.

#### Thickness, wet weight, and volume measurement

IVEC thickness was measured using a Vernier caliper, and wet weight was determined with an electronic balance. For volume measurements, samples were immersed in 5 ml of absolute ethanol, and the volume was calculated based on the observed displacement.

#### Histological and immunohistochemical evaluation of IVEC

IVEC samples from each group were fixed in 4% paraformaldehyde for 24 hours, embedded in paraffin and sectioned into 5-μm slices. H&E staining was performed to assess the histological structure, while Safranin-O staining was used to evaluate GAG deposition. Immunohistochemistry was conducted to detect type II collagen (COL II) expression.

#### Quantification of cartilage-specific proteins

Cartilage-specific protein content was quantified by analyzing GAG levels using the DMMB assay, and COL II content was measured using an ELISA kit (Nanjing Jiancheng, China).

#### Biomechanical analysis

The Young’s modulus of IVEC samples was determined using a biomechanical analyzer (Instron-5542, Canton, USA). Samples were subjected to continuous unconfined compression at a strain rate of 1 mm/min until reaching 80% of maximum deformation. The Young’s modulus was calculated from the slope of the resulting stress-strain curve [26].

### Biological effects of different culture systems

#### Live/dead cell viability staining

To evaluate cell viability within the IVEC across different culture systems, Live/dead cell viability staining (Invitrogen, USA) was performed on days 1, 5 and 9 of culture according to the manufacturer’s instructions. Cell survival was observed using a confocal microscope (Leica, Germany) [27].

#### Quantification of cell viability and DNA content

Cell viability was quantified using the CCK-8 assay as described above. DNA content was measured using the PicoGreen dsDNA fluorescence assay.

#### ELISA

Samples from each culture system were homogenized to release extracellular proteins. After centrifugation, the supernatants were collected and analyzed by ELISA to quantify lysyl oxidase (LOX) and pyridinoline (PYR) using goat-specific ELISA kits.

#### Expression of cartilage-related pathway proteins

Western blotting was performed to analyze the expression of LOX, Wnt/β-catenin, TGF-β/Smad and MAPK pathway-related proteins in chondrocytes cultured under different systems. Approximately 50–200 mg of frozen cartilage tissues were minced and homogenized in 500 µl of ice-cold RIPA lysis buffer supplemented with 1× protease inhibitor cocktail (RIPA: PIC: PMSF = 100:1:1, v/v/v) using a tissue homogenizer. The homogenate was incubated on ice for 30 minutes, followed by centrifugation at 12 000 × g for 30 minutes at 4°C. The resulting supernatant containing total protein extracts was carefully collected for subsequent analysis. Protein concentrations were determined using a BCA Protein Assay Kit. Equal amounts of protein were separated by SDS-PAGE, transferred to PVDF membranes, and blocked with TBST. The membranes were then incubated overnight at 4°C with primary antibodies (diluted 1:1000) against LOX, Wnt/β-catenin, TGF-β/Smad, and MAPK. After three washes with TBST, the membranes were incubated with secondary antibodies (diluted 1:2000) for 30 minutes, followed by additional washes. β-actin was used as an internal control, and protein bands were visualized using a chemiluminescent substrate. Quantitative analysis of the images was conducted using ImageJ (NIH, USA).

### Cartilage regeneration via subcutaneous implantation of IVEC in a goat model

#### Subcutaneous implantation of IVEC into a goat

Ethical approval for this study was obtained from the Hainan Medical University Ethics Committee. The surgical site on the dorsum of the goat (from which auricular cartilage was obtained as described in the section Isolation and culture of goat auricular chondrocytes) was disinfected, and an incision was made. Blunt dissection was performed to create subcutaneous pouches. IVEC samples from the three groups (as described in section Maturity evaluation of IVEC under different culture systems) were implanted into distinct pouches within the same goat. The incision was then sutured and disinfected, with strict aseptic techniques maintained throughout the procedure. Postoperative monitoring included regular assessments of the goat’s general condition and the surgical site for signs of infection or graft exposure. Samples were harvested at 4 and 8 weeks post-implantation.

#### Evaluation of in vivo regenerated cartilage

Retrieved samples (with surface fibrous connective tissue carefully removed) were first photographed using a digital camera for macroscopic examination. The freshly obtained cartilage tissues were then subjected to a mechanical analyzer (Instron-5542, Canton, USA) without undergoing pre-conditioning, hydration, fixation or freezing procedures. Mechanical properties (*n* = 3 per group) were assessed through unconfined compressive testing. Continuous planar unconfined compression tests were performed at room temperature at a constant speed of 1 mm/min, corresponding to a strain rate of 0.033 ± 0.0021/s, until sample failure (indicated by the inflection point on the force–displacement curve). A biomechanical analyzer recorded force–displacement data in real time. The compressive Young’s modulus was derived from these curves for subsequent statistical analysis [28].

Portions of the samples were then sectioned and subjected to histological staining, including H&E, Safranin-O and COL II immunohistochemical staining, to evaluate the tissue structure and cartilage-specific matrix deposition. For collagen II detection, tissue sections were subjected to heat-induced antigen retrieval in sodium citrate buffer (pH 6.0, 95°C, 20 minutes), followed by PBS washes. After peroxidase blocking (3% H_2_O_2_, 10 minutes, RT) and PBS rinses, sections were blocked with 10% normal sheep serum (37°C, 30 minutes) and incubated with anti-collagen II primary antibody (1:100, 4°C overnight). Following rewarming (37°C, 30 minutes) and washing, sections were treated with HRP-conjugated secondary antibody (1:2000, 37°C, 1 hour). Color development used DAB/H_2_O_2_ (5–10 minutes) with microscopic monitoring, followed by hematoxylin counterstaining and standard mounting procedures.

The thickness of the engineered cartilage was measured with a micrometer, and the wet weight was determined using an electronic balance via the water displacement method.

Samples were minced and digested overnight with papain. Sulfated GAG content was quantified using the Alcian blue assay (Sigma-Aldrich, USA), and COL II content was measured by ELISA (Sigma-Aldrich, USA).

### Statistical analysis

Statistical analysis was performed using SPSS version 19.0. Data from at least three independent experiments are presented as mean ± standard deviation (SD). Group differences were analyzed using a *t*-test and/or one-way analysis of variance, followed by Tukey’s post hoc test for multiple comparisons. Statistical significance was set at *P* < 0.05.

## Results

### Preparation of DWJM

**Figure 1.**
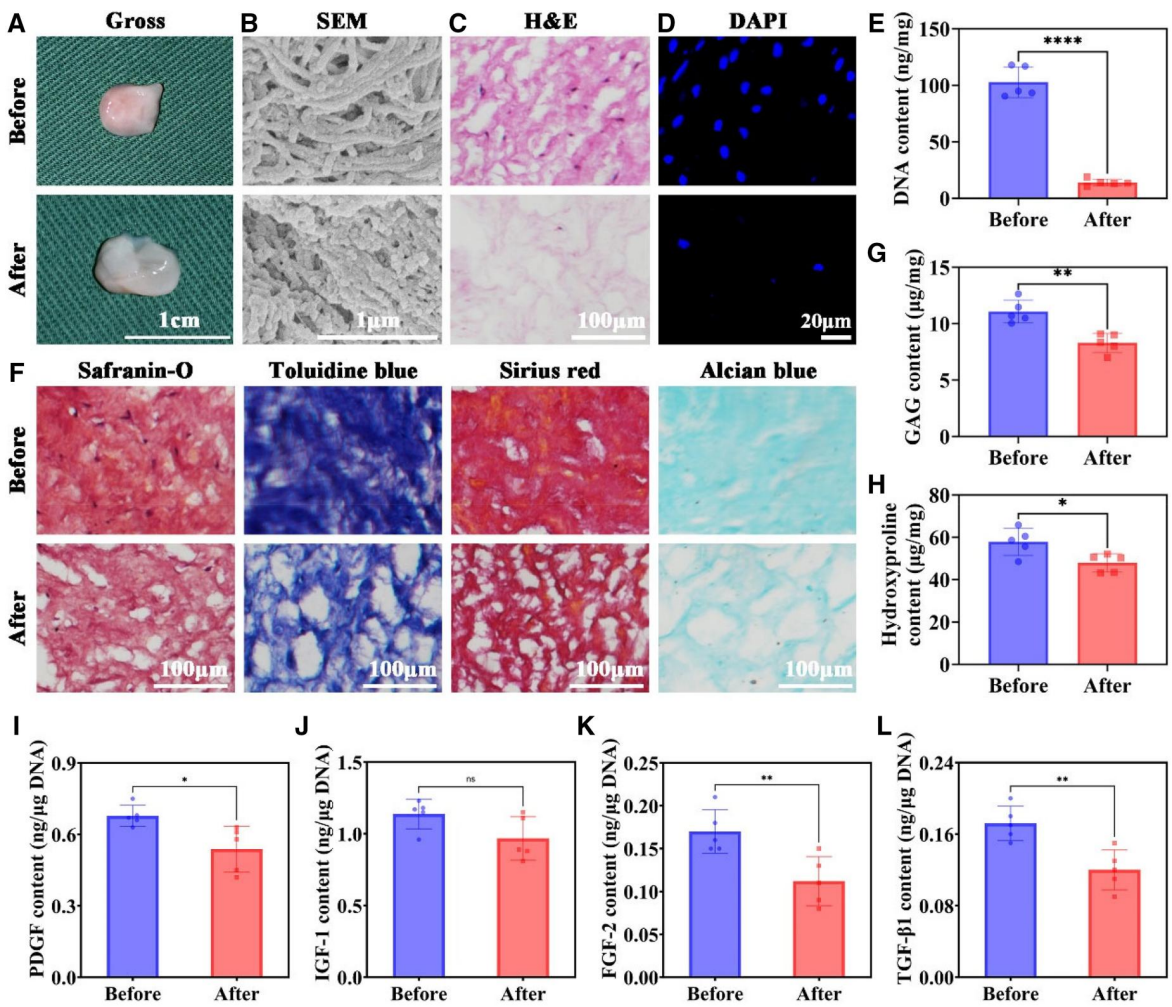
Decellularization of WJ. (**A–D**) Gross, SEM, H&E staining and DAPI staining for WJ before and after decellularization. (**E**) DNA content quantification for WJ before and after decellularization. (**F**) Safranin-O, toluidine blue, sirius red and Alcian blue stainings for WJ before and after decellularization. Quantitative analysis of biochemical components in cartilage, including (**G**) GAG and (**H**) hydroxyproline content for WJ before and after decellularization. Quantitative analysis of bioactive factors related to chondrogenic induction, including (**I**) PDGF content, (**J**) IGF-1 content, (**K**) FGF-2 content and (**L**) TGF-β1 content for WJ before and after decellularization. *P < 0.05, **P < 0.01, ****P < 0.0001, ns indicates no statistically significant difference.

The original WJ exhibited a gelatinous appearance with a white base tinted with red. After decellularization, the resulting DWJM appeared white, translucent, and had a softer texture with slight deformation (Figure 1A). SEM images revealed that although the continuous fibrous network of WJ was partially disrupted by decellularization, the overall fibrous structure was retained (Figure 1B). H&E staining demonstrated that the ECM became loosened and most nuclei were removed following decellularization (Figure 1C). DAPI staining further confirmed the near-complete removal of nuclei, which was corroborated by quantitative DNA analysis (Figures 1D and E). Special histological stains—including Safranin-O, Toluidine blue, Sirius red, and Alcian blue—showed that while the original WJ was rich in GAGs, proteoglycans, collagen and chondroitin sulfate, DWJM retained the majority of these components despite exhibiting a looser structure and decreased cellular content (Figure 1F). Quantitative analyses of GAG and hydroxyproline indicated a partial reduction in cartilage-specific ECM components (Figures 1G and H), and measurements of bioactive factors revealed that although PDGF, IGF-1, FGF-2 and TGF-β1 levels were partially reduced after decellularization, most factors were preserved (Figure 1I–L). Collectively, these data confirm the successful preparation of DWJM with retention of most cartilage-related ECM components and bioactive factors.

### Synthesis of photo-crosslinkable ADWJM hydrogels

**Figure 2.**
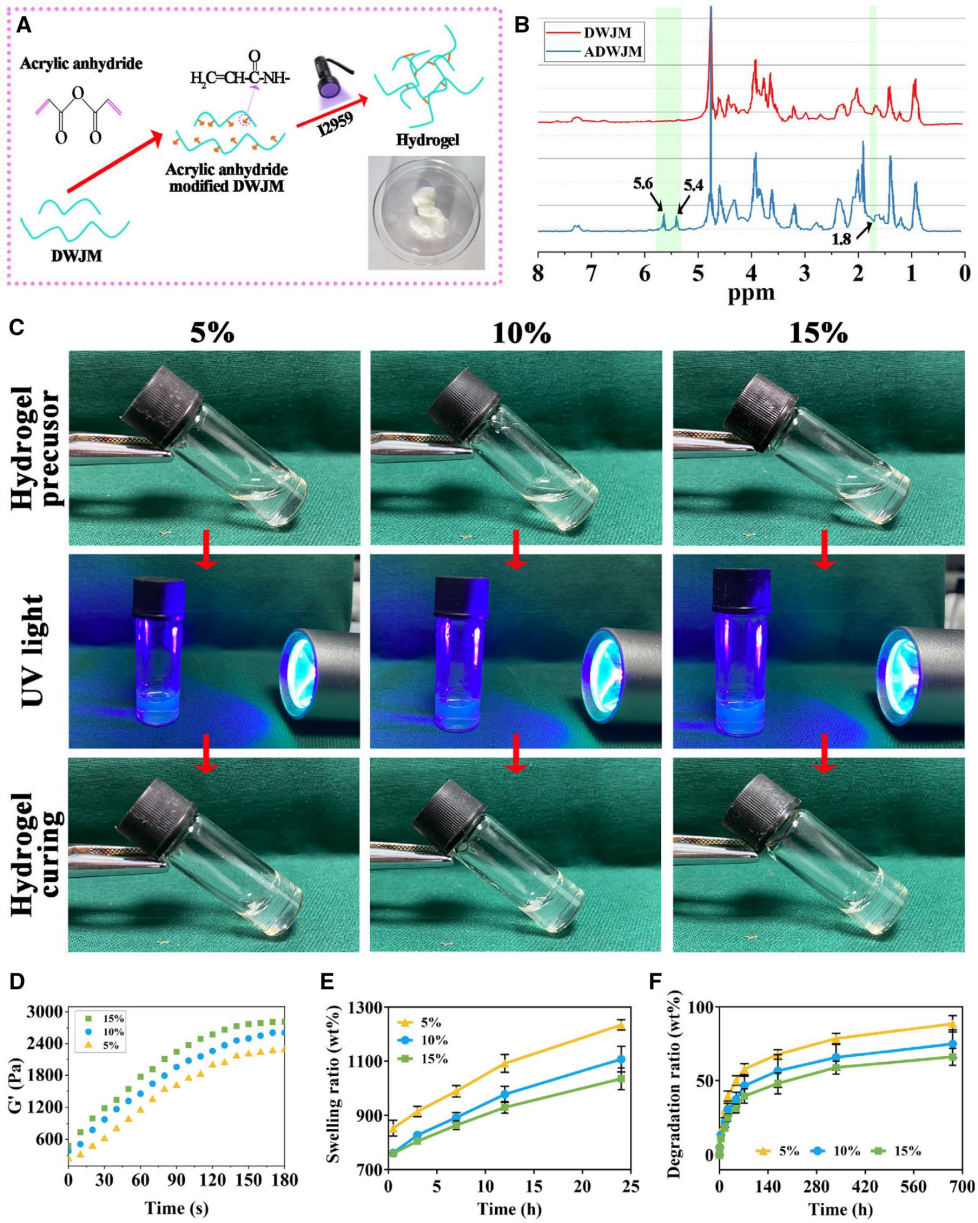
Preparation and characterizations of photo-crosslinkable ADWJM hydrogels. (**A**) Schematic illustration of the preparation of photo-crosslinkable ADWJM hydrogels using acrylated DWJM. (**B**) ^1^H NMR spectrum for DWJM and ADWJM. (**C**) Hydrogel precursors could be gelated upon UV light at concentrations of 5%, 10%, and 15%. (**D**) G′ (storage modulus), (**E**) swelling ratio and (**F**) degradation ratio of ADWJM hydrogels at the three different concentrations.

DWJM was modified with methacrylate anhydride to produce photo-crosslinkable ADWJM hydrogels at concentrations of 5%, 10% and 15% (w/v) (Figure 2A). The ^1^H NMR spectrum of ADWJM exhibited two prominent vinyl proton peaks at 5.4 and 5.6 ppm and a small methyl peak at 1.8 ppm, confirming the successful grafting of methacrylate groups onto the WJ molecular chains (Figure 2B). Upon 10 minutes of UV exposure, the ADWJM hydrogel precursors solidified across all concentrations (Figure 2C). Rheological time-sweep analysis demonstrated that the hydrogels possessed a robust storage modulus (G′) that increased with higher DWJM concentrations, indicating a shift toward stronger elastic behavior (Figure 2D). Although swelling and degradation rates increased over time in all groups, the higher-concentration hydrogels exhibited lower rates, suggesting improved stability (Figure 2E and F).

### Biocompatibility of ADWJM hydrogels to chondrocytes

**Figure 3.**
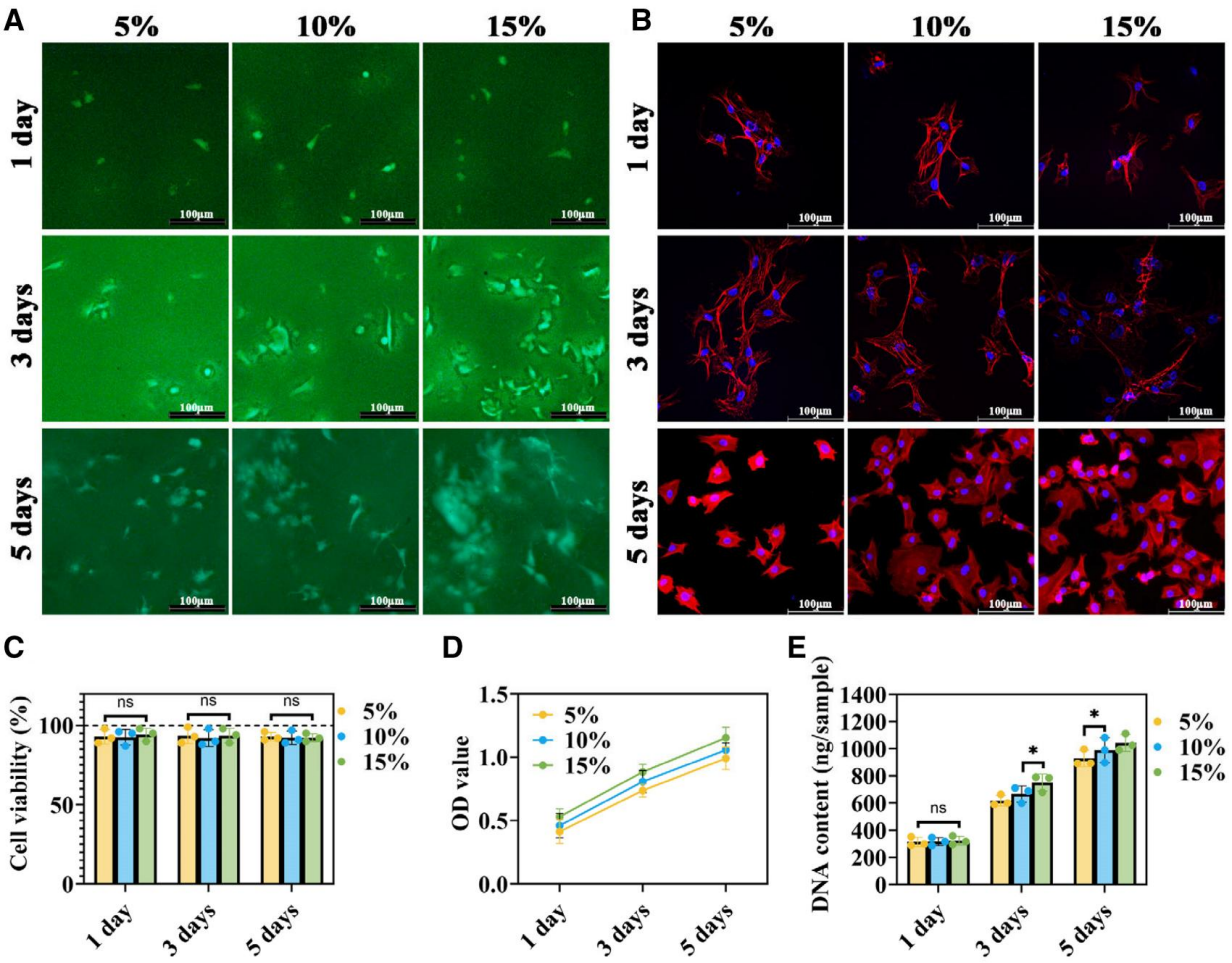
Biocompatibility evaluation of ADWJM hydrogel to chondrocytes. (**A**) Live/dead staining for ADWJM hydrogels in different concentrations when cocultured with chondrocytes at 1, 3 and 5 days. (**B**) F-actin/DAPI staining for ADWJM hydrogels in different concentrations when cocultured with chondrocytes at 1, 3 and 5 days. Quantitative analysis of (**C**) cell viability, (**D**) OD values and (**E**) DNA content in various groups at 1, 3 and 5 days. *P < 0.05, ns indicates no statistical significance.

The biocompatibility of ADWJM hydrogels (5%, 10% and 15% w/v) was evaluated by co-culturing them with chondrocytes. Live/dead staining over a 5-day period revealed an increasing number of green-stained live chondrocytes in all groups, with only a few red-stained dead cells observed; notably, the 15% hydrogel supported the greatest number of live cells (Figure 3A). Cytoskeletal staining demonstrated that chondrocytes were well spread in all hydrogels and proliferated more extensively over time, particularly at higher DWJM concentrations (Figure 3B). CCK-8 assays confirmed nearly 100% cell viability across all groups, with OD values increasing in both a time- and concentration-dependent manner—again, with the 15% group showing the highest values (Figure 3C and D). DNA quantification further indicated a significant increase in DNA content with prolonged culture time and higher DWJM concentrations (Figure 3E). Overall, these results demonstrate that ADWJM hydrogels are highly biocompatible and exhibit low cytotoxicity. Based on optimal cell viability and structural stability, the 15% formulation was selected for subsequent *in vitro* and *in vivo* cartilage regeneration experiments.

### Maturity evaluation of IVEC

**Figure 4.**
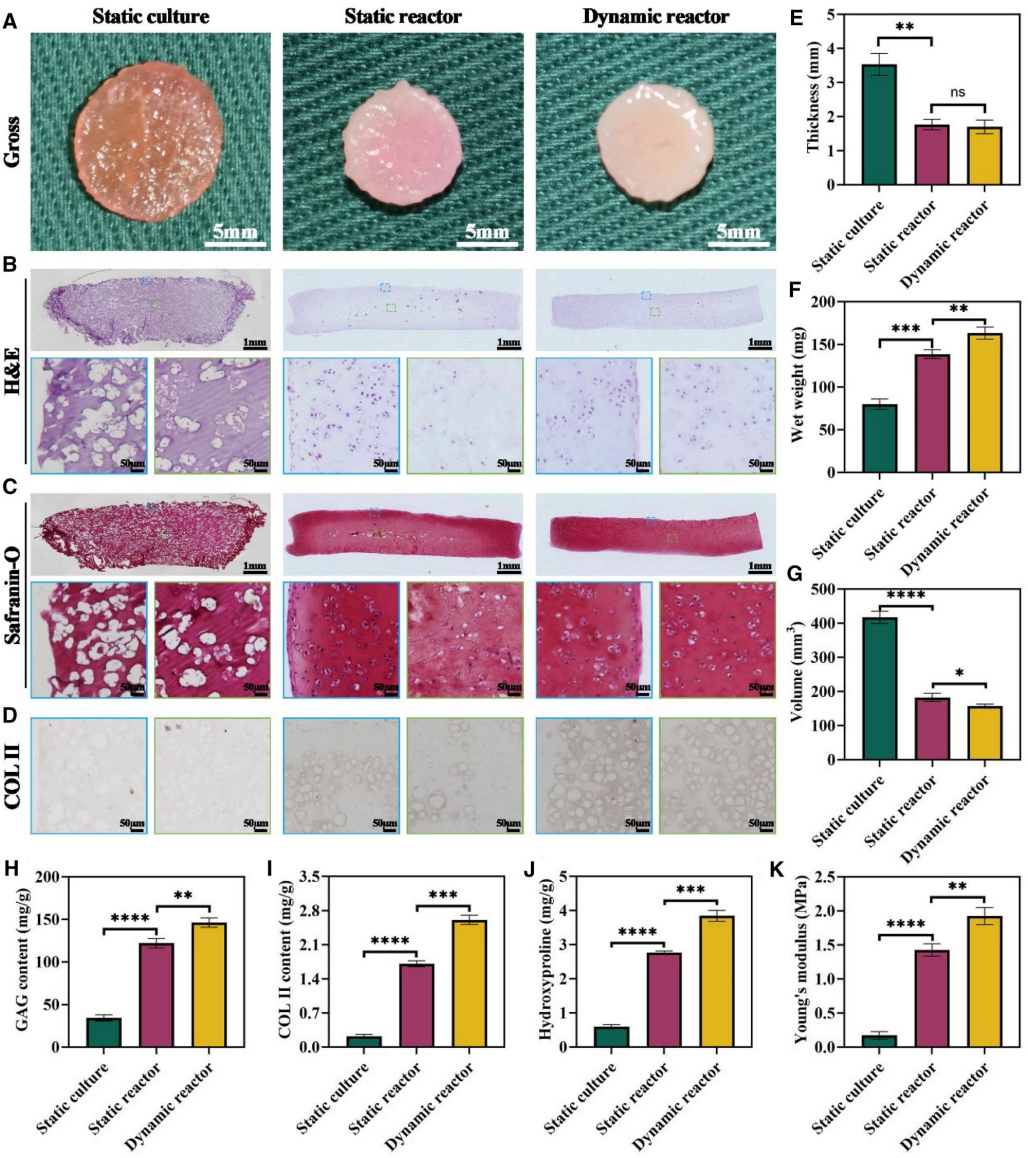
*In vitro* evaluation of cartilage regeneration in dynamic reactor. (**A**) Macroscopic view for samples from static culture, static reactor and dynamic reactor groups after 4 weeks *in vitro* culture. (**B–D**) Histological staining of regenerated cartilage under different culture systems, including H&E, Safranin-O and immunohistochemical COL II stainings. Blue boxes indicate the peripheral region, while green boxes denote the central region. (**E–G**) Quantitative analysis of cartilage thickness, wet weight and volume in regenerated cartilage under different culture systems. (**H–J**) Measurement of extracellular matrix components, including GAG, COL II and hydroxyproline, in regenerated cartilage under various culture systems. (**K**) Biomechanical analysis of Young’s modulus in regenerated cartilage under different culture systems. *P < 0.05, **P < 0.01, ***P < 0.001, ****P < 0.0001, ns indicates no statistical significance.

To determine the effect of different culture systems on IVEC maturity, chondrocyte–hydrogel constructs (using the 15% ADWJM formulation) were cultured for 4 weeks under static conditions, in a static bioreactor, or in a dynamic bioreactor. Macroscopically, samples from the dynamic bioreactor group maintained their original shape and exhibited a smooth, dense, ivory-white appearance. The static bioreactor group retained its shape and showed a pink appearance with a relatively smooth surface, although its texture was inferior to that of the dynamic group. In contrast, the static culture group underwent significant deformation, displaying a soft, pink and rough appearance (Figure 4A).

Histological and immunohistochemical analyses revealed that the static culture group exhibited minimal cartilage-like features, with poorly defined lacunae and negligible ECM deposition. The static bioreactor group formed noticeable cartilage-like features with clear lacunae and positive ECM deposition in peripheral regions, although central regions were heterogeneous. In contrast, the dynamic bioreactor group developed uniform cartilage-like tissue, characterized by abundant lacunae and extensive cartilage-specific ECM deposition throughout both peripheral and central areas (Figure 4B–D).

Quantitative analyses further demonstrated that constructs from the dynamic bioreactor group had significantly greater thickness, wet weight, and volume compared to those from the static bioreactor and static culture groups (Figure 4E–G). Similarly, levels of cartilage-specific ECM components—including GAG, COL II and hydroxyproline—were highest in the dynamic bioreactor group, intermediate in the static bioreactor group, and lowest in the static culture group (Figure 4H–J). Biomechanical testing showed that the dynamic bioreactor group achieved the highest Young’s modulus (Figure 4K). Together, these findings indicate that dynamic bioreactor culture markedly promotes the maturation of IVEC.

### Biological effects of IVEC under different culture systems

**Figure 5.**
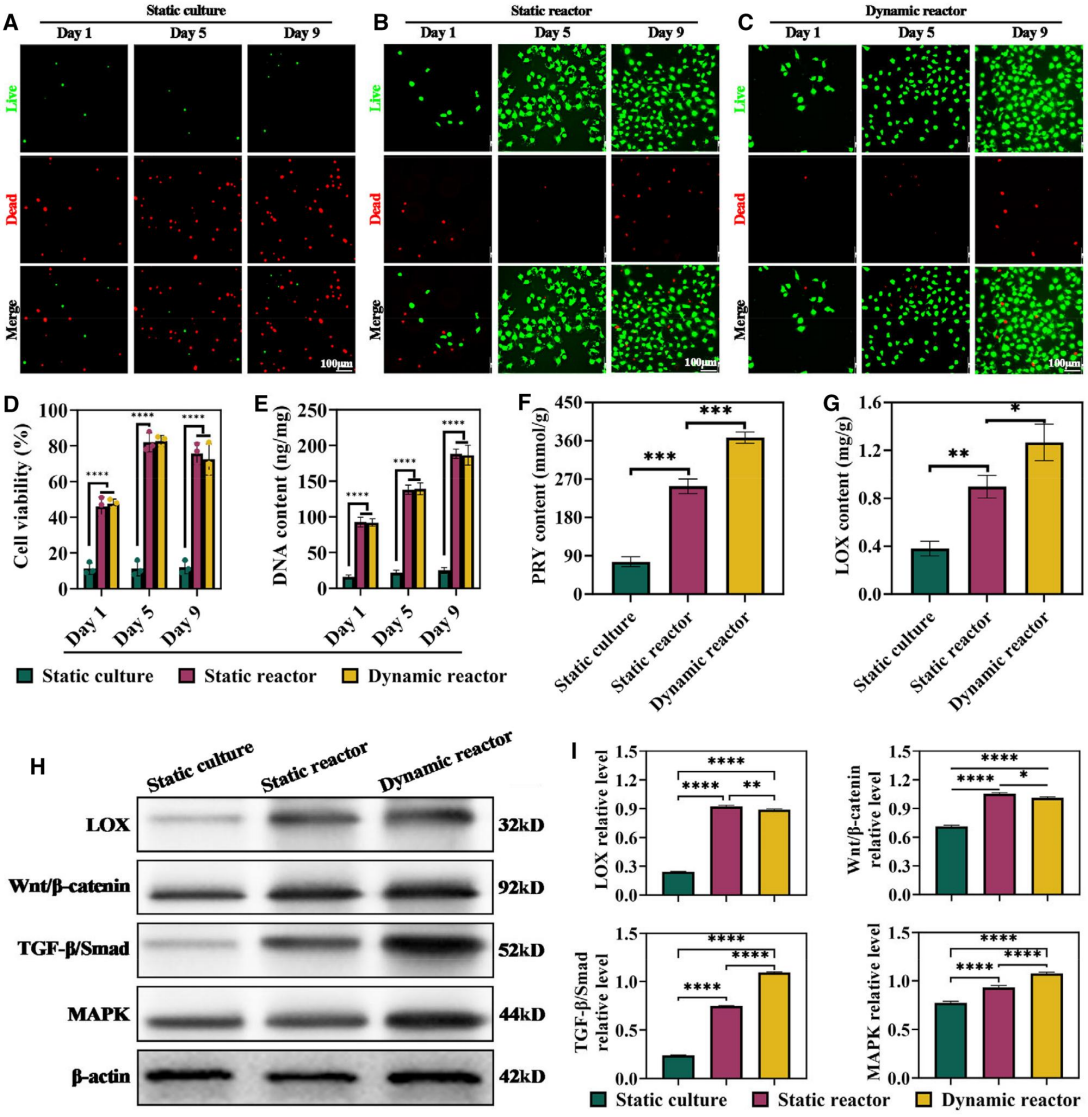
Biological effects of *in vitro* regenerated cartilage under different culture systems. (**A–C**) Live/dead staining of chondrocytes under different culture systems at day 1, 5 and 9, where green indicates live cells and red indicates dead cells. (**D**) Chondrocyte viability in different culture systems at day 1, 5 and 9, as determined by the CCK-8 assay. (**E**) DNA content of chondrocytes in different culture systems at day 1, 5 and 9. (**F** and **G**) Quantification of PYR and LOX contents in chondrocytes under different culture systems. (**H**) Expression of pathway-related proteins in chondrocytes in different culture systems, including LOX, Wnt/β-catenin, TGF-β/smad and MARK. (**I**) Quantitative analysis of LOX, Wnt/β-catenin, TGF-β/smad and MARK protein expression levels in different culture systems. *P < 0.05, **P < 0.01, ***P < 0.001, ****P < 0.0001.

Live/dead staining over a 9-day period revealed extensive cell death in the static culture group, whereas chondrocytes in both static and dynamic bioreactor systems maintained robust proliferation (Figure 5A–C). These observations were confirmed by CCK-8 assays and DNA quantification, which demonstrated significantly higher viability and DNA content in the bioreactor groups compared to the static culture group (Figure 5D–E). Moreover, quantitative analyses showed that the dynamic bioreactor group produced the highest levels of PYR and LOX, followed by the static bioreactor group, with the static culture group exhibiting the lowest levels (Figure 5F–G). Western blot analysis further revealed that expression levels of LOX, as well as key proteins in the Wnt/β-catenin, TGF-β/Smad and MAPK pathways, were significantly higher in the dynamic bioreactor group than in the other groups (Figure 5H and I).

### Cartilage formation via subcutaneous implantation of IVEC in a goat model

**Figure 6.**
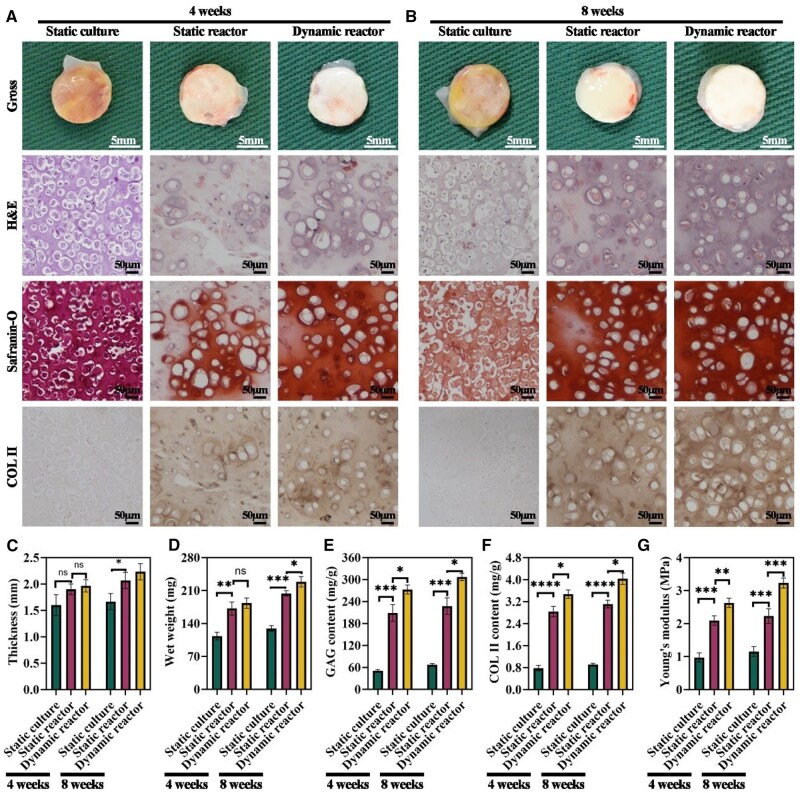
Evaluation of cartilage formation via subcutaneously implantation of *in vitro* regenerated cartilage under different culture systems in a goat model. (**A**) Gross appearance, H&E staining, Safranin-O staining and immunohistochemical COL II staining of cartilage tissue after 4 weeks of subcutaneously implantation. (**B**) Gross appearance, H&E staining, Safranin-O staining and immunohistochemical COL II staining of cartilage tissue after 8 weeks of subcutaneously implantation. (**C** and **D**) Thickness and wet weight of *in vivo* regenerated cartilage under different culture systems at 4 and 8 weeks. (**E** and **F**) Quantitative analysis of GAG and COL II contents in *in vivo* regenerated cartilage under different culture systems at 4 and 8 weeks. (**G**) Biomechanical analysis of Young’s modulus in *in vivo* regenerated cartilage under different culture systems at 4 and 8 weeks. *P < 0.05, **P < 0.01, ***P < 0.001, ****P < 0.0001, ns indicates no statistical significance.

To evaluate *in vivo* maturation, 4-week-old IVEC constructs from the different culture systems were implanted subcutaneously into a goat. After 4 weeks of implantation, samples from the dynamic bioreactor group exhibited a porcelain-like color and smooth texture, closely resembling native cartilage. The static bioreactor group also presented a cartilage-like appearance with a pink hue and relatively smooth surface, whereas the static culture group displayed pink, fibrous characteristics (Figure 6A). At 8 weeks post-implantation, both the static and dynamic bioreactor groups developed more typical cartilage-like features; notably, the dynamic bioreactor group most closely approximated mature cartilage. In contrast, the static culture group failed to generate cartilage-like tissue even after extended implantation (Figure 6B).

Histological staining (H&E, Safranin-O) and COL II immunohistochemistry further confirmed these observations. After 8 weeks, the static culture group lacked cartilage-specific ECM deposition and lacunae, while both the static and dynamic bioreactor groups showed clear cartilage-specific ECM deposition and well-defined lacunae. Importantly, the dynamic bioreactor group exhibited more uniform lacunae and stronger positive staining for Safranin-O and COL II (Figure 6A and B). Quantitative measurements revealed that constructs from the dynamic bioreactor group had significantly greater thickness, wet weight, and higher levels of GAG, COL II, and Young's modulus compared with the static bioreactor and static culture groups (Figure 6C–G). Moreover, both the static and dynamic bioreactor groups showed improvements in these parameters from the pre-implantation state (after 4 weeks *in vitro*) to 8 weeks post-implantation, indicating that *in vivo* conditions further promote IVEC maturation.

## Discussion

Hydrogel‐based IVEC offers clear advantages for repairing cartilage defects *in vivo*, yet current methods struggle to produce adequately mature tissue. In this study, we developed a novel photo-crosslinkable ADWJM hydrogel—combined with dynamic bioreactor culture—to enhance IVEC maturation. First, WJ was decellularized to obtain DWJM, effectively eliminating immunogenic cellular components while preserving key ECM molecules. The DWJM was then modified with methacrylate anhydride to produce a photo-crosslinkable hydrogel with ideal gelation properties, adjustable rheological behavior, controlled swelling ratios, and predictable degradation rates that can be tuned by varying DWJM concentration. Notably, chondrocyte‐laden ADWJM hydrogels cultured in a dynamic bioreactor achieved superior maturation compared to those maintained in static bioreactors or under static culture conditions. Furthermore, subcutaneous implantation in a goat model yielded more mature *in vivo* cartilage, demonstrating the clinical potential of our approach.

The reproducible fabrication of functionally mature hydrogel-based IVEC continues to pose significant translational challenges, particularly in recapitulating the complex biomechanical and biochemical niche required for chondrocyte maturation. Our previous studies demonstrated that hydrogels derived from methacrylate anhydride-modified WJ could reconstruct C-shaped tracheal cartilage rings in animal models [22]. However, those hydrogels did not yield satisfactory IVEC, likely because of intrinsic limitations. Hydrogels are highly hydrated 3D networks that, upon swelling, experience a reduction in pore size [29]. This can impede cell migration, restrict nutrient diffusion, slow waste removal and ultimately impair cell proliferation and ECM synthesis—problems that are exacerbated in thicker constructs [30]. Traditional strategies, such as the addition of bioactive factors to hydrogels, are limited by high costs, short durations of action and inconsistent efficacy [23, 31, 32]. Consequently, it is critical to identify more efficient hydrogels and culture methods for the clinical translation of cartilage tissue engineering.

WJ is an attractive source for cartilage engineering because it contains few cells yet is rich in cartilage‐related ECM components (e.g. GAGs, proteoglycans, collagen and chondroitin sulfate) and peptide growth factors (e.g. PDGF, IGF‐1, FGF‐2 and TGF‐β1) that promote chondrogenesis [33, 34]. These components provide a favorable microenvironment for cartilage regeneration by supporting chondrocyte proliferation and differentiation. Our results confirm that WJ is abundant in these factors, making it an ideal biomaterial for IVEC.

Decellularized ECM scaffolds have been demonstrated to recapitulate key features of native ECM, including microstructure, biochemical composition and biological function [35, 36]. We employed a NaOH-based decellularization process that effectively removed cellular components while preserving the WJ’s bioactive constituents. The combination of mechanical mincing and controlled NaOH treatment allowed for the dissolution of cytoplasmic elements, nucleic acids and non-crosslinked proteins without compromising the integrity of the collagen matrix [37–39]. By fine-tuning parameters such as NaOH concentration, treatment duration and temperature, we achieved high-quality DWJM that retains most of its inherent bioactive factors, providing a solid foundation for subsequent hydrogel synthesis.

The DWJM was then modified with methacrylate anhydride to produce a photo-crosslinkable ADWJM hydrogel. Increasing the DWJM concentration produced a denser 3D network with higher storage modulus (G′), reduced swelling ratios and slower degradation rates [40, 41]. Our results demonstrated that ADWJM hydrogels provide an excellent microenvironment for chondrocyte encapsulation, survival and proliferation—exhibiting outstanding biocompatibility and low cytotoxicity. Moreover, increasing the concentration of DWJM resulted in a greater number of viable chondrocytes. This effect can be attributed to two factors. First, a higher DWJM content increases the presence of native ECM components and adhesion sites, thereby supplying abundant biochemical cues and a microenvironment that closely mimics native tissue. Second, a denser crosslinking network—characterized by an increased storage modulus, reduced swelling ratio and slower degradation rate—provides continuous, stable and compliant 3D support. Together, these factors synergistically enhance chondrocyte viability and activity during *in vitro* culture.

Despite these benefits, hydrogel-based IVEC typically exhibits lower maturity than natural cartilage, primarily due to suboptimal culture environments [42]. Identifying suitable culture conditions is therefore a critical focus. Bioreactors, with their capabilities for automation, precise control, and real-time monitoring, have been successfully applied in osteochondral tissue engineering [43]. Dynamic compression, for instance, has been shown to boost cartilage regeneration by increasing COL II and GAG content [44]. The dynamic bioreactor in our study simulates the physiological conditions of natural cartilage by precisely controlling both mechanical stimulation and fluid dynamics. Its mechanisms of action include the following: (i) activating intracellular signaling pathways (such as Wnt/β-catenin, TGF-β/Smad and MAPK) via dynamic compression and shear stress to promote chondrocyte proliferation and ECM synthesis; (ii) ensuring efficient nutrient and oxygen delivery while facilitating waste removal, thus maintaining a homogeneous cell culture environment; (iii) enhancing cell adhesion, migration and matrix remodeling to preserve the structural integrity of the developing cartilage; (iv) integrating mechanical and biochemical signals to further optimize the microenvironment for cartilage regeneration. Collectively, these mechanisms provide ideal conditions for IVEC maturation, advancing cartilage tissue engineering and laying a theoretical and practical foundation for future *in vivo* cartilage repair.

In our study, we compared the maturity of IVEC under three different culture conditions: dynamic bioreactor, static bioreactor and static culture. The dynamic bioreactor group produced the thickest, most uniform and most stable cartilage tissue *in vitro*. This finding aligns with previous reports that bioreactors facilitate the real-time exchange of nutrients and waste between hydrogels and culture media, thereby promoting chondrocyte adhesion, synchronized maturation across the construct and maintenance of tissue shape [45]. In contrast, IVEC cultured under static conditions was markedly inferior, and even the static bioreactor group showed better maturation only in peripheral regions. We speculate that the dynamic bioreactor more effectively mimics *in vivo* mechanical and nutritional environments, delivering physical and chemical stimuli that enable IVEC to closely resemble natural cartilage in both structure and function [46, 47]. Our data further revealed that the dynamic bioreactor group exhibited the highest PYR and LOX contents, suggesting enhanced collagen crosslinking and improved mechanical properties [15]. Additionally, continuous dynamic stimulation activated LOX, Wnt/β-catenin, TGF-β/Smad and MAPK signaling pathways in chondrocytes more robustly than in static systems, thereby promoting collagen synthesis, ECM strength, chondrocyte proliferation and differentiation, and ultimately, collagen fiber maturation [48–50].

The capacity of IVEC to continue growing after *in vivo* implantation is a critical clinical concern. Our results show that after implantation in a goat, IVEC from the dynamic bioreactor group formed more uniform and mature cartilage compared to those from static culture and static bioreactor groups. Post-implantation, we observed increases in tissue thickness, wet weight, cartilage ECM content and Young’s modulus relative to the pre-implantation state. Moreover, these parameters were significantly higher in the dynamic bioreactor group than in the static bioreactor group. We attribute the improvement in *in vivo* cartilage regeneration to the enhanced IVEC produced under dynamic bioreactor conditions. The observed thickness reduction in static-cultured constructs may be attributed to hydrogel degradation, cellular condensation-induced volumetric compaction and physiological stress adaptation. These findings collectively suggest that static culture conditions alone might not adequately support optimal *in vivo* cartilage regeneration when using hydrogel-based IVEC. Consequently, dynamic culture conditions appear more conducive to cartilage ECM deposition and tissue maturation, offering promising solutions for the challenges associated with hydrogel-based IVEC.

We conducted additional analysis to thoroughly compare the mechanical properties of our dynamically cultured IVEC constructs with those of native cartilage. Specifically, we assessed the Young's modulus of the engineered constructs under dynamic bioreactor conditions, obtaining values ranging approximately 3.2 MPa. These values align closely with reported Young’s modulus values for native cartilage tissue (3–5 MPa) [51]. This similarity underscores the effectiveness of our dynamic cultivation approach combined with ADWJM hydrogels in achieving biomechanical properties analogous to natural cartilage. Additionally, we recognize the importance of evaluating mechanical integrity as a crucial criterion for successful clinical translation. Hence, the achieved mechanical performance suggests that our engineered constructs are capable of withstanding physiological mechanical stresses comparable to native cartilage, reinforcing the potential of this approach for clinical applications.

Despite these promising findings, several limitations remain. First, the use of autologous chondrocytes may cause donor site morbidity; future studies should consider alternative cell sources such as stem cells derived from bone marrow, adipose tissue, or skin. Second, the precise mechanisms by which dynamic bioreactors activate chondrocyte signaling pathways require further investigation. Third, we evaluated only three DWJM concentrations (5%, 10% and 15%); additional studies are needed to optimize the hydrogel composition. Finally, further refinement of bioreactor parameters is essential to shorten the cartilage maturation process and improve scalability for clinical applications.

## Conclusion

In summary, our study demonstrates that a photo-crosslinkable ADWJM hydrogel, combined with dynamic bioreactor culture, significantly enhances the maturity of IVEC both *in vitro* and *in vivo*. This approach offers a promising strategy for advancing cartilage tissue engineering and overcoming current limitations in hydrogel-based cartilage repair.

## Funding

This research was supported by Hainan Province Clinical Medical Center, Hainan Provincial Natural Science Foundation (ZDYF2020130), and Natural Science Foundation of China (82360560).

*Conflicts*  *of interest statement*. None declared.

## Data Availability

The datasets generated during and/or analyzed during the current study are available from the corresponding author on reasonable request.
